# Mechanosynthesis and Thermoelectric Properties of Fe, Zn, and Cd-Doped P-Type Tetrahedrite: Cu_12-x_M_x_Sb_4_S_13_

**DOI:** 10.3390/ma14133448

**Published:** 2021-06-22

**Authors:** Francisco Arturo López Cota, José Alonso Díaz-Guillén, Oscar Juan Dura, Marco Antonio López de la Torre, Joelis Rodríguez-Hernández, Antonio Fernández Fuentes

**Affiliations:** 1 Cinvestav Unidad Saltillo, Apartado Postal 663, Saltillo 25000, Coahuila, Mexico; 2 División de Estudios de Posgrado e Investigación, Instituto Tecnológico de Saltillo, Saltillo 25280, Coahuila, Mexico; j.a.diazguillen@gmail.com; 3 GFMA, Departamento de Física Aplicada, Escuela Técnica Superior de Ingenieros Industriales, Universidad de Castilla-La Mancha, 13071 Ciudad Real, Spain; oscar.juan@uclm.es (O.J.D.); marcoantonio.lopez@uclm.es (M.A.L.d.l.T.); 4 Centro de Investigación en Química Aplicada, Saltillo 25294, Coahuila, Mexico; joelis.rodriguez@ciqa.edu.mx

**Keywords:** thermoelectric, mechanosynthesis, tetrahedrites, Seebeck, thermal conductivity, figure of merit, electrical conductivity

## Abstract

This contribution deals with the mechanochemical synthesis, characterization, and thermoelectric properties of tetrahedrite-based materials, Cu_12-x_M_x_Sb_4_S_13_ (M = Fe^2+^, Zn^2+^, Cd^2+^; x = 0, 1.5, 2). High-energy mechanical milling allows obtaining pristine and substituted tetrahedrites, after short milling under ambient conditions, of stoichiometric mixtures of the corresponding commercially available binary sulfides, i.e., Cu_2_S, CuS, Sb_2_S_3_, and MS (M = Fe^2+^, Zn^2+^, Cd^2+^). All the target materials but those containing Cd were obtained as single-phase products; some admixture of a hydrated cadmium sulfate was also identified by XRD as a by-product when synthesizing Cu_10_Cd_2_Sb_4_S_13_. The as-obtained products were thermally stable when firing in argon up to a temperature of 350–400 °C. Overall, the substitution of Cu(II) by Fe(II), Zn(II), or Cd(II) reduces tetrahedrites’ thermal and electrical conductivities but increases the Seebeck coefficient. Unfortunately, the values of the thermoelectric figure of merit obtained in this study are in general lower than those found in the literature for similar samples obtained by other powder processing methods; slight compositional changes, undetected secondary phases, and/or deficient sintering might account for some of these discrepancies.

## 1. Introduction

Thermoelectric materials have gained in recent years special relevance in multiple energy-related applications, such as power generation or waste heat recovery [1,2]. Using the Seebeck effect, thermoelectric materials convert a temperature gradient into a voltage difference and thereby transform thermal energy directly into electricity (and vice versa). Their efficiency is assessed by a dimensionless quantity known as the thermoelectric figure of merit, which is represented by (*zT*) and given by the mathematical expression (*zT*) = *σS*^2^*T*/*κ*, where *S* is the Seebeck coefficient, *σ* represents the electrical conductivity, *T* is the absolute temperature and *κ* is the thermal conductivity. Moreover, the ideal thermoelectric material (high (*zT*) values) would be one having a high electrical conductivity and Seebeck coefficient but low thermal conductivity [3]; unfortunately, finding candidate materials meeting these criteria has proven very difficult because the three (*S*, *σ*, and *κ*) are coupled, and any attempt to optimize one affects the remaining two.

Commonly available thermoelectric materials at present include Bi_2_Te_3_, PbTe-based materials, and Si-Ge alloys; however, increasing concerns about the cost, long-term availability, and environmental impact of some of their constituent chemical elements have fueled the search for more benign alternatives [4,5]. Among them, Cu_12_Sb_4_S_13_-based tetrahedrites have been identified as a very promising group of low-cost and environmentally friendly thermoelectric materials. This mixed valence Cu^+^/Cu^2+^ sulfide features an isometric unit cell (Space Group $I\bar{4}3m$ (#217)) with a single Sb site, two equally populated Cu sites, and two S positions in a rather complex crystal structure with a large number of atoms per unit cell (Z = 2); furthermore, tetrahedrites can accommodate a large variety of Cu and Sb substitutions, display p-type semiconducting behavior, and have intrinsically low thermal conductivity, which are all desirable characteristics for a thermoelectric material. Their ample chemical and structural flexibility allow for the tuning of their properties for different energy-related applications such as supercapacitors, photovoltaics, or catalysis, to mention some [6,7,8,9]. Herein, we describe a simple solid-state method to prepare pure and substituted tetrahedrites Cu_12-x_M_x_Sb_4_S_13_ (M = Cd, Zn and Fe), using binary sulfides as starting materials. Metal sulfides are generally prepared in laboratory, by firing either under vacuum or in oxygen-free environments, stoichiometric powdered mixtures of high-purity sulfur and the corresponding metal(s) or by complex and lengthy solution-mediated methods [10,11,12]. Alternatively, they have been also obtained from the elements by dry or liquid-assisted mechanochemical methods under controlled atmospheres, or through a mechanically induced chemical reaction between an alkali metal sulfide and the corresponding metal acetates [13,14,15,16]. The synthesis of tetrahedrite-based mixed sulfides by reacting volatile sulfur (b.p. = 717.8 K) and high melting point Cu (m.p. = 1357.8 K) is particularly complex, and it must be carried out in evacuated and sealed glass ampoules to minimize sulfur losses and ensure single-phase products with controlled stoichiometries. Furthermore, the synthesis is hindered by the difficulties in controlling the final oxidation state of copper, which should be present as mixed Cu(I)/Cu(II). This article describes an alternative method for obtaining tetrahedrites from commercially available binary sulfides, using high-energy mechanical milling under ambient conditions. Mechanochemically obtained materials are frequently highly defective and metastable phases, featuring a large variety of structural defects such as vacancies, antisite defects, and dislocations [17]. Defects might be beneficial for suppressing the materials’ thermal conductivity and thus for increasing the thermoelectric figure of merit [18]. The proposed methodology might be a feasible high-throughput route for producing different complex chalcogenides, even using naturally occurring and relatively abundant sulfide minerals, as starting chemicals.

## 2. Materials and Methods

The synthesis of the title materials was carried out by high-energy mechanical milling, using commercially available binary sulfides (i.e., Cu_2_S, CuS, Sb_2_S_3_, FeS, ZnS, and CdS) as starting chemicals. As-received reactants (Sigma-Aldrich Inc., St. Louis, MO, USA, ≥99%) were weighed out as required by stoichiometry to obtain the corresponding Cu_12-x_M_x_Sb_4_S_13_ (M = Cd, Zn, and Fe) materials and then hand mixed in an agate mortar and placed in 125 mL YPSZ containers (5 mol% Y_2_O_3_) together with 20 mm diameter YPSZ balls as grinding media (ball/powder mass ratio = 10:1). Milling was performed under ambient conditions (air) in a Retsch PM400 planetary mill (Retsch GmbH, Haan, Germany), using a rotating sun-wheel speed of 350 rpm (vial-to-wheel speed ratio 1:−2). The evolution of the reacting mixtures with milling time was followed by sampling the content of the jar at different time intervals (30 min, 1, 3, and 6 h), and analyzing the as-obtained powders by X-ray diffraction (XRD) (Rigaku Ultima IV, Rigaku Co, Tokyo, Japan). All mechanically induced chemical reactions were considered completed when no traces of the starting powders were identified by this technique.

### Characterization

For the structural analysis, XRD patterns were collected at room temperature using CuKα radiation (typical scan rate = 2° min^−1^, in 0.02° (2θ) steps) and a Rigaku Ultima IV diffractometer equipped with a D/teX detector. Structural refinement of the XRD data was performed by using the Rietveld method, implemented in the FULLPROF program [19]. Cell parameters and peak profiles were refined using Le Bail’s pattern fitting method and a pseudo-Voigt peak shape function [20], whereas the background was modeled by a third-order polynomial fitting. Reference structural data for tetrahedrite were obtained from the Inorganic Crystal Structure Database [21]. The thermal stability of the as-obtained powders was analyzed by recording simultaneously their weight change and heat flow (TGA/DTA) between room temperature and 550 °C using a TA Instruments SDT 600 Analyzer (TA Instruments, New Castle, DE, USA) (typical sample size 5 mg; flowing argon = 100 mL/min; heating rate = 10 °C/min).

Thermal properties were analyzed in pellets (≈0.5 cm thickness and 1.3 cm diameter) prepared by uniaxially pressing (5 ton/cm^2^) and firing (350 °C; soaking time = 1 h) the mechanochemically obtained mixed sulfides. Thermal diffusivities (α) were determined by the laser pulse technique (inert atmosphere), with a commercial LINSEIS LFA 1000 equipment (Linseis Messgeräte GmbH, Selb, Germany). Before every measurement, samples were coated with a thin layer of colloidal graphite to ensure full absorption of the energy pulse at the pellet’s front side and the highest emissivity from the back side. Samples and coating stability were confirmed by performing three consecutive analyses under identical conditions. Heat capacity was determined with the help of a simultaneous DSC/TGA Jupiter Netzsch STA 449C equipment (Netzsch Holding, Selb, Germany) in the temperature range from 50 to 350 °C (Pt crucibles; heating rate = 10 °C/min; an argon flow = 35 mL/min). A sapphire single crystal sample was used as reference material. As before, several runs were carried out for each sample to verify our experimental data reproducibility and accuracy.

The Seebeck coefficient was measured in the temperature range from 50 to 350 °C (under vacuum; heating rate = 5 °C/min) in an MMR-Technologies SB-100 instrument (MMR Technologies Inc, Mountain View, CA, USA), taking three readings every 25 °C. Resistivity measurements were made at the same conditions as the Seebeck coefficient, using a Keithley 2400 Source Meter (Tektronix Inc, Beaverton, OR, USA).

## 3. Results and Discussion

### 3.1. X-ray Diffraction (XRD)

**Figure 1:**
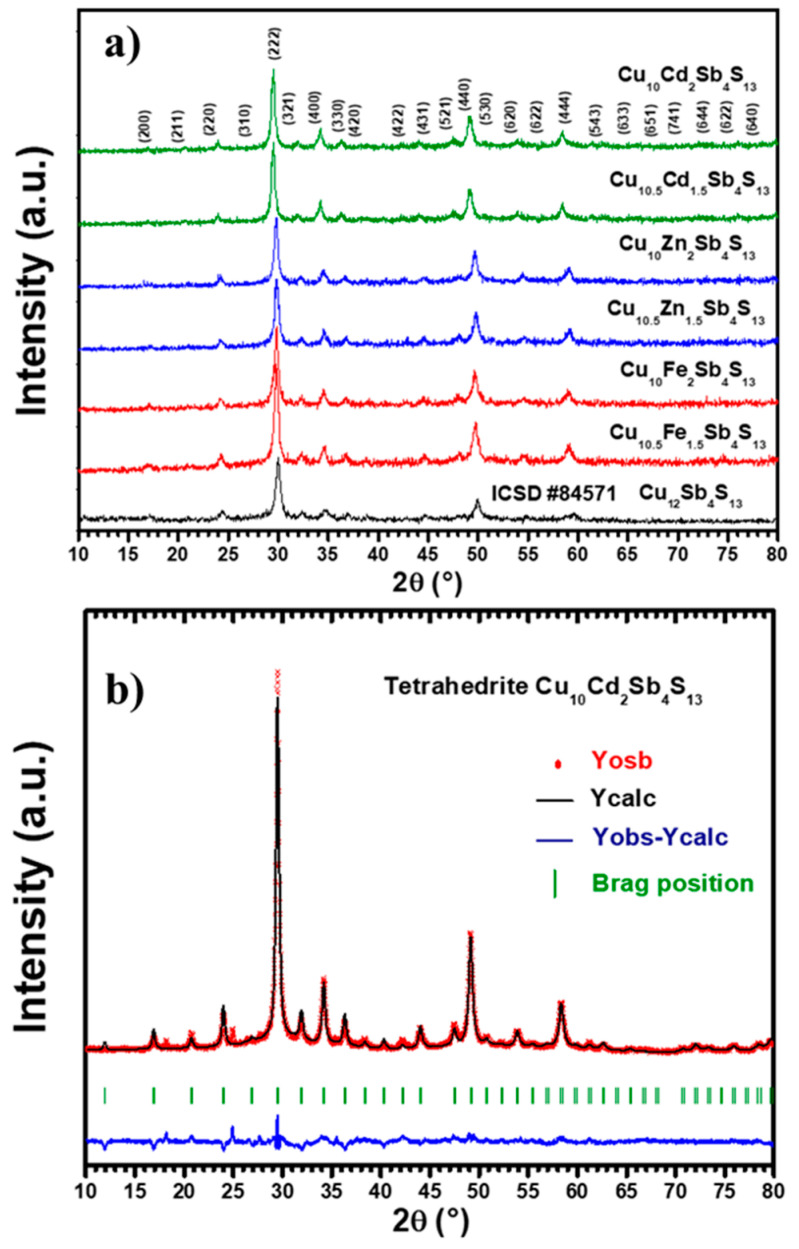
(**a**) XRD patterns for Cu_12-x_M_x_Sb_4_S_13_ (x = 1.5, 2) compared with the standard data ICSD #84571. (**b**) Rietveld refinement graph of Cu_10_Cd_2_Sb4S_13_.

Figure 1a shows a comparison between the XRD patterns collected for the as-obtained Cu_12_Sb_4_S_13_ and Cu_12-x_M_x_Sb_4_S_13_ samples (x = 1.5, 2; M = Fe^2+^, Cd^2+^, and Zn^2+^), after milling the corresponding reacting mixtures for 6 h, as described in the previous section. All reacting mixtures present after this milling time an XRD pattern similar to that described in the literature as characteristic of cubic tetrahedrite. Accordingly, the numbers in parentheses in Figure 1a are the corresponding Miller indexes, as stated in ICSD #84571. The purity and room-temperature crystallographic characteristics of the as-prepared sulfides were analyzed by XRD using the Rietveld method and the corresponding crystal data from the literature. Figure 1b shows the graphic result of the refinement carried out in the Cu_10_Cd_2_Sb_4_S_13_ sample, whereas Table 1 presents the results and reliability factors for the whole series. Fe and Zn-containing mixtures produced single-phase products, whereas the XRD pattern collected for the sample with the highest Cd content (Cu_10_Cd_2_Sb_4_S_13_) showed in Figure 1b also contains two additional and very low intensity reflections at 18 and 25° (2θ). These reflections suggest the existence of a small admixture of CdSO_4_·H_2_O as secondary phase. The crystal chemistry of copper-based sulfides is rather complex and determined to a great extent by the S/Cu atomic ratio [22]. Thus, all Cu atoms are found in almost regular tetrahedral coordination when Cu/S << 1, but in irregular geometries with lower coordination numbers, when the ratio is close to or greater than 1. In tetrahedrite (Cu/S = 0.92), half of all Cu atoms occupy the Wyckoff 12d tetrahedral site (coordination polyhedra CuS_4_), and the other half sits at the Wyckoff 12e site, adopting a rather unusual trigonal planar coordination (coordination polyhedra CuS_3_); substituting divalent metals replacing Cu are limited to two atoms per formula unit (0 ≤ *x* ≤ 2) and occupy preferentially the tetrahedral site. According to the results summarized in Table 1, all the samples prepared in this work were successfully refined using the tetrahedrite crystal structure [16,23]. Depending on the substituent element, our results show that they have cell parameters ranging from 10.366 to 10.462 Å, similar to those reported in the literature for partially substituted tetrahedrites [24,25]. The largest change, which is evidenced in the diffraction pattern with a small shift toward higher angles (2θ), corresponds to the tetrahedrite where 100% of CuS was replaced by CdS; this change is mostly due to the ionic radius of cadmium, which shows the largest mismatch to that of Cu.

### 3.2. Thermal Analysis

**Figure 2:**
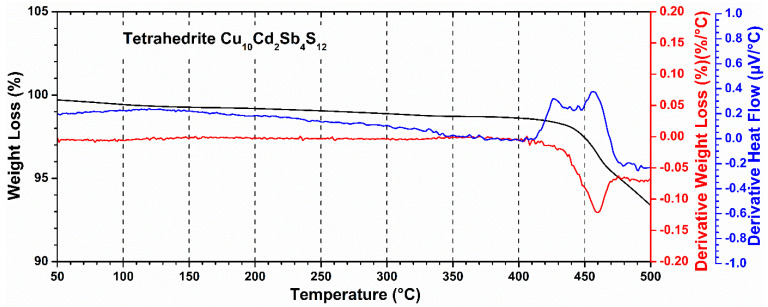
Weight loss, derivative weight loss, and derivative of DTA for phase Cu_10_Cd_2_Sb4S_13_.

A series of thermal analysis studies were carried out to identify the thermal stability of these materials, which is important when determining their maximum operating temperature. In Figure 2, it is possible to observe the percentage of weight loss, the derivative weight loss, and the derivative heat flow for the reference sample Cu_10_Cd_2_Sb_4_S_13_, which was selected as representative for the series. The material obtained presents thermal stability until 400 °C, determining this as the operating range. The maximum weight loss of the composition starts at a temperature of 460 °C, which is probably due to sulfur loss. However, it was decided to measure the electrical and thermal properties in a range of 50 to 350 °C, since the rest of the phases decomposed at lower temperatures.

### 3.3. Thermal Conductivity

To determine the thermal properties of the material, it was necessary to measure each of the components that make up the figure of merit. The first to be measured was the Cp inherent in the samples studied; Table 2 shows the heat capacity of the different phases studied at a temperature of 350 °C. Likewise, the phase tetrahedrite without substitution was used as a reference. From the table, it is appreciated that all the phases present similar Cp values.

**Figure 3:**
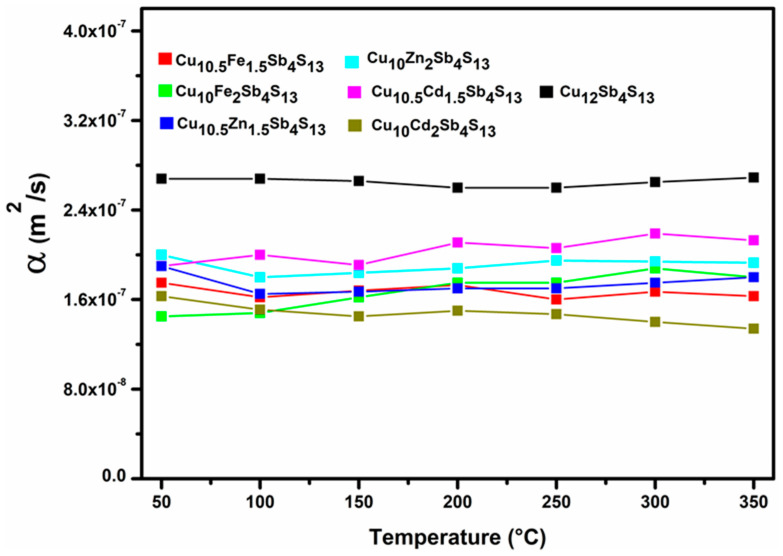
Behavior of thermal diffusivity for the unsubstituted tetrahedrite phase and for the mono-substituted tetrahedrites, in a temperature range between 50 and 350 °C.

Figure 3 shows the thermal diffusivity of each phase studied. It is evident that the unsubstituted tetrahedrite has a higher thermal diffusivity (2.69 × 10^−7^ m^2^/s) than the remaining tetrahedrites. Of the mono-substituted compounds, the one with the highest diffusivity values is the sample Cu_10.5_Cd_1.5_Sb_4_S_13_ with 2.13 × 10^−7^ m^2^/s, while the one with the lowest levels is the sample Cu_10_Cd_2_Sb_4_S_13_ 1.34 × 10^−7^ m^2^/s.

**Figure 4:**
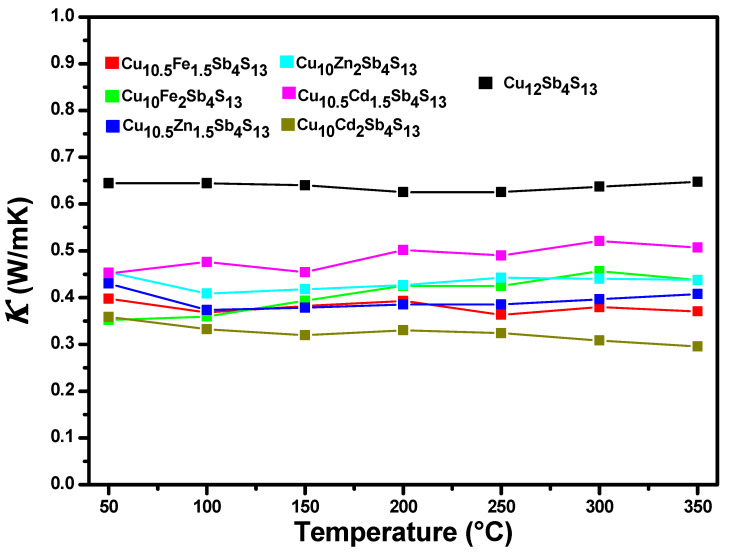
Behavior of thermal conductivity for the tetrahedrite phase without substitution and for the mono-substituted tetrahedrites.

The thermal conductivity for each of the mono-substituted samples as well as that of the unsubstituted tetrahedrite are presented in Figure 4; the sample that presents the highest thermal conductivity is the tetrahedrite without substitution (0.65 W/m K), while the rest of the samples present lower levels of conductivity between 0.35 and 0.5 W/m K. The heat capacity is similar for all these materials, as well as their density, since they have the same chemical base only with small variations, being the thermal diffusivity the one that implies a drastic change at the moment of obtaining the thermal conductivity of the material. The total thermal conductivity has two major contributions: the lattice part, which accounts for the propagation of phonons through the crystalline lattice, and the electronic part (*κ_e_*) accounting for the transport of heat with charger carriers. The electronic part of the thermal conductivity was calculated using the Wiedemann–Franz law as *κ_e_* = *L*_0_*σT*, where *L*_0_ is the ideal Lorenz number, 2.44 × 10^−8^ WΩK^−2^, σ is the electrical conductivity, and *T* is the absolute temperature. The electronic part of the thermal conductivity is very low and accounts for no more than 1% of the total thermal conductivity [26]. The decrease in thermal conductivity for the mono-substituted samples may be due to point defects generated with the incorporation of substituents to the crystal lattice or to dislocations produced during the mechanical grinding of the material.

### 3.4. Power Factor

**Figure 5:**
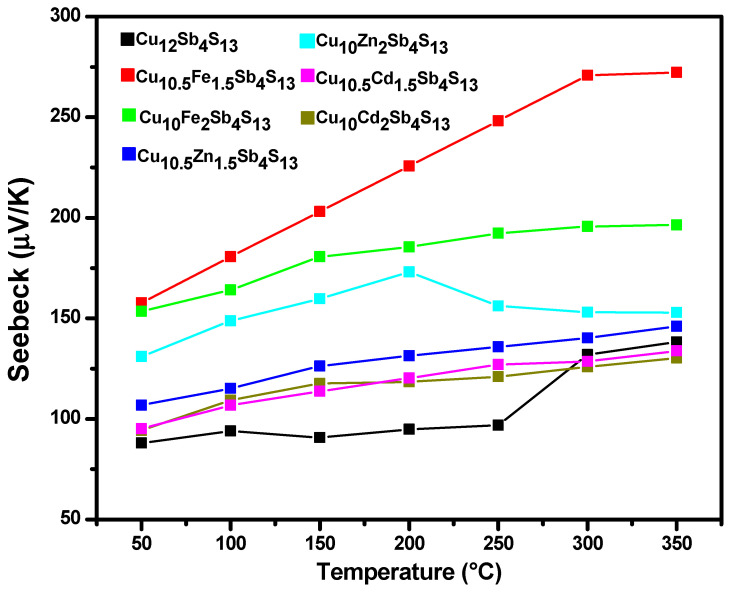
Seebeck coefficient for the tetrahedrite phase without substitution and for the mono-substituted tetrahedrites.

The first element analyzed that integrates the power factor into the figure of merit is the Seebeck coefficient, which can be observed in Figure 5 for each studied sample. The highest values obtained for the Seebeck coefficient correspond to the compound Cu_10.5_Fe_1.5_Sb_4_S_13_, while tetrahedrite without substitution was the sample with the lowest coefficient in almost every single temperature. Every single result is similar or even higher than those reported [27]. Most of the compounds show a general behavior of between 130 and 272 µV/K. The results of this measurement agree even with tetrahedrites of mineral origin, which indicates a similarity of the synthetic phase with the natural tetrahedrite [28]. The positive behavior in the Seebeck coefficient indicates that they are p-type semiconductors indicating that majority carriers are holes, forming a network that will allow an electronic flow through it. 

**Figure 6:**
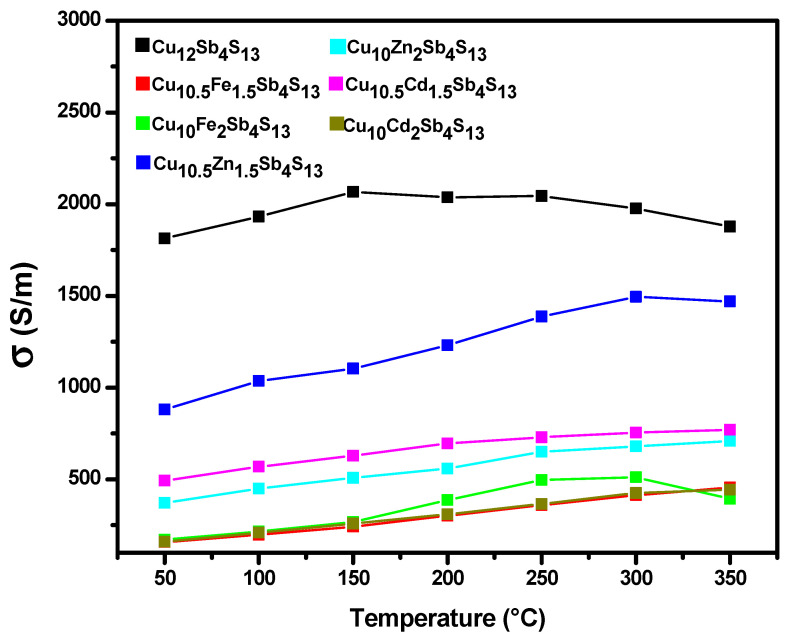
Electrical conductivity in pristine tetrahedrite and in mono-substituted Cu_12-x_M_x_Sb_4_S_13_ (M = Cd, Fe, Zn) in a temperature range between 50 and 350 °C.

The electrical conductivity that is inverse to the resistivity was analyzed for each compound and can be seen in Figure 6. Tetrahedrite in its base form is the one with the highest conductivity value with 1877 S/m, while the samples corresponding to the Cu_10.5_Fe_1.5_Sb4S_13_, Cu_10_Cd_2_Sb_4_S_13_, and Cu_10_Fe_2_Sb_4_S_13_ phases are the ones with the lowest levels with 456 S/m, 443 S/m, and 394 S/m, respectively. The results agree with other thermoelectric materials studied and are synthesized with fast techniques such as the mechanical milling [29]. This behavior is possibly because the flow of electric current is hampered by encountering dislocations caused by mechanical grinding. However, the substitution by other elements increases the Seebeck coefficient and decreases the thermal conductivity, which is beneficial to increasing the figure of merit. The higher the value obtained for conductivity, the greater the number of positive charges.

**Figure 7:**
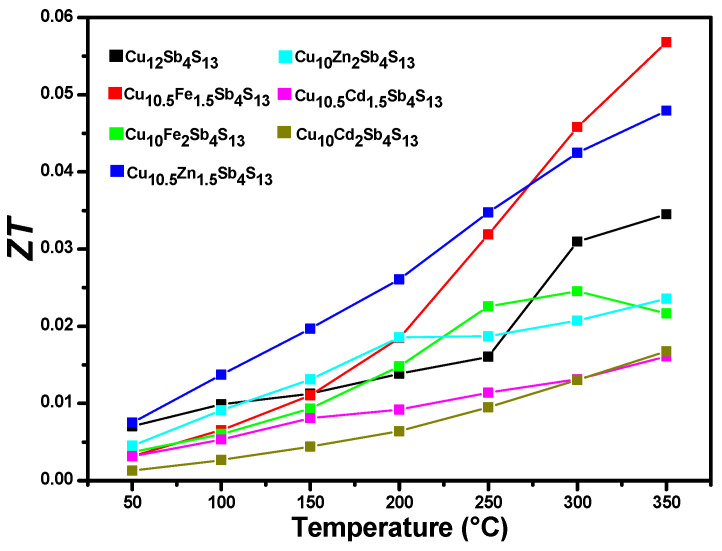
Figure of merit of pristine tetrahedrite and the substituted Cu_12-x_M_x_Sb_4_S_13_-type tetrahedrite family.

With the results of the thermal and electrical properties, it is possible to obtain the figure of merit for each compound. Figure 7 shows the zT values for the analyzed mono-substituted samples as well as the base tetrahedrite. The materials with formula Cu_12-x_M_x_Sb_4_S_13_ that present the highest zT values are Cu_10.5_Fe_1.5_Sb_4_S_13_ with zT = 0.057, followed by Cu_10.5_Zn_1.5_Sb_4_S_13_ zT = 0.048. The first one has a low electrical conductivity of 456 S/m; however, its Seebeck coefficient is the highest (7.41 × 10^−8^), while the second one has the highest electrical conductivity of the substituted samples with 1470 S/m. Tetrahedrite in its base form has a zT = 0.035 despite its poor thermal properties; however, it is the sample that presents the highest electrical conductivity results with 1877 S/m. zT values obtained for tetrahedrite-based samples vary widely depending on factors such as the synthesis and sintering conditions, dopant, and doping levels; furthermore, samples having theoretically a similar chemical composition but obtained by different research groups using similar procedures yield different zT values. Thus, values reported for pristine Cu_12_Sb_4_S_13_ include 0.13 at 400 K [30], 0.52 at 600 K [31], 0.76 at 623 K [27], or 0.65 at 723 K [32]. According to the literature, zT values for Fe-doped tetrahedrites, Cu_12-x_Fe_x_Sb_4_S_13_, are very sensible to Fe content and temperature, with the highest values obtained when x ≤ 1 and the temperature higher than 700 K. Thus, some reported values are 0.01 (at 340 K), 0.6 (550 K), and 0.8 (720 K) measured for respectively, Cu_10_Fe_2_Sb_4_S_13_ [33], Cu_11_FeSb_4_S_13_ [34], and Cu_11.5_Fe_0.5_Sb_4_S_13_ [35]. Zn-doped tetrahedrites have been also studied, yielding zT values of 0.03 at 340 K [33], 0.038 at 573 K [36], or even 1.0 at 723 K [35]. As for Cd-doping, zT values as high as 0.9 at 623 K have been reported for Cu_11.25_Cd_0.75_Sb_4_S_13_ [27]. In general, the values found in this study are lower than those mentioned above. Slight compositional changes, undetected secondary phases, and residual porosity might account for some of these discrepancies. The results of density, diffusivity, and thermal and electrical conductivity measurements to obtain the figure of merit of each material from this work are listed in Table 3.

## 4. Conclusions

It is possible to obtain Cu_12_Sb_4_S_13_-type materials by mechanical grinding as well as the partial or total substitution of covellite in the tetrahedrite phase without the need for the use of inert atmospheres employing short synthesis times starting from a mixture of high-purity commercial reagents. This also showed that the electrical and thermal properties obtained are similar or even bigger than those reported for thermoelectric materials in this temperature range.

## Figures and Tables

**Table 1:** Experimental details for the XRD data recording and processing.

Compound	Cu_10.5_Cd_1.5_Sb_4_S_13_	Cu_10_Cd_2_Sb_4_S_13_	Cu_10.5_Zn_1.5_Sb_4_S_13_	Cu_10_Zn_2_Sb_4_S_13_	Cu_10.5_Fe_1.5_Sb_4_S_13_	Cu_10_Fe_2_Sb_4_S_13_
ICSD	84571					
**Unit Cell**						
Space group	I-43m	I-43m	I-43m	I-43m	I-43m	I-43m
Cell parameters (Å)	a = 10.4627(10)	a = 10.4870(9)	a = 10.3540(9)	a = 10.3667(9)	a = 10.3562(13)	a = 10.3662(12)
V(Å^3^)	1145.33(2)	1153.33(2)	1110.01(2)	1114.09(2)	1110.71(2)	1113.93(2)
Z	2	2	2	2	2	2
**Refinement**						
Number of reflections	51	51	51	51	51	51
Number of refined parameters						
Structural	9	9	9	9	9	9
Profile	8	8	8	8	8	8
Rexp	1.85	1.87	1.88	1.9	1.86	1.96
Rwp	3.98	3.81	3.84	3.92	3.68	3.7
R_B_	2.33	2.26	2.22	2.23	2.1	2.13
S	2.15	2.04	2.04	2.06	1.97	1.89

**Table 2:** Heat capacity for mono-substituted tetrahedrites at 350 °C.

	Tetrahedrites	Cp J/kg K
Pristine	Cu_12_Sb_4_S_13_	515.99
Mono-substituted	Cu_10.5_Fe_1.5_Sb_4_S_13_	524.29
	Cu_10_Fe_2_Sb_4_S_13_	567.043
	Cu_10.5_Zn_1.5_Sb_4_S_13_	548.63
	Cu_10_Zn_2_Sb_4_S_13_	522.67
	Cu_10.5_Cd_1.5_Sb_4_S_13_	559.22
	Cu_10_Cd_2_Sb_4_S_13_	495.12

**Table 3:** Results corresponding to the parameters of thermal and electrical conductivity to obtain the figure of merit.

	Tetrahedrites	ρ (kg/m^3^)	α (m^2^/s)	κ (W/m K)	*S* (µV/K)	σ (S)	zT
Pristine	Cu_12_Sb_4_S_13_	4662.162	2.69 × 10^−7^	0.647	1.91 × 10^−8^	1876.89	0.035
Mono-substituted	Cu_10_Fe_1.5_Cu_0.5_Sb_4_S_13_	4333.333	1.63 × 10^−7^	0.370	7.41 × 10^−8^	455.52	0.057
	Cu_10_Fe_2_Sb_4_S_13_	4283.582	1.80 × 10^−7^	0.437	3.86 × 10^−8^	394.32	0.022
	Cu_10_Zn_1.5_Cu_0.5_Sb_4_S_13_	4127.45	1.80 × 10^−7^	0.408	2.13 × 10^−8^	1469.59	0.048
	Cu_10_Zn_2_Sb_4_S_13_	4342.857	1.93 × 10^−7^	0.438	2.34 × 10^−8^	708.66	0.024
	Cu_10_Cd_1.5_Cu_0.5_Sb_4_S_13_	4255.319	2.13 × 10^−7^	0.507	1.70 × 10^−8^	769.99	0.016
	Cu_10_Cd_2_Sb_4_S_13_	4448.275	1.34 × 10^−7^	0.295	1.79 × 10^−8^	443.26	0.017

## Data Availability

These results are part of F.A.L.C. Ph.D. project carried out at Cinvestav del IPN, Unidad Saltillo, Mexico.
